# Continuity of care in Danish peer-led patient online communities on social media. A survey study

**DOI:** 10.1093/eurpub/ckac131.294

**Published:** 2022-10-25

**Authors:** L Ledderer, A Karlsson, C Stage

**Affiliations:** 1 Institute of Public Health, Aarhus University, Aarhus, Denmark; 2 School of Communication and Culture, Aarhus University, Aarhus, Denmark

## Abstract

**Background:**

Patients in the Danish healthcare system and other Western countries experience a lack of ‘continuity of care’ due to inadequate communication and sharing of clinical information. ‘Continuity of care’ is often defined as informational, managerial and relational failures. A lack of continuity is particularly problematic for patients with chronic conditions as they are often highly dependent on longitudinal and complex treatment processes. Some of these patients have formed self-organised groups on social media in order to share their personal experiences with the health care system and discuss health related problem with peers. The aim of this paper is to understand the role peer-led online communities (PLOC) play for patients with chronic conditions experiences of continuity of care.

**Methods:**

The material consists of survey data from patients with chronic conditions participating in peer-led online communities on the experience of continuity of care in the Danish healthcare system. A link to the survey was posted in the public online community “Chronic Influencers” (Instagram 10,000 followers), and in the closed Facebook group “Chronic pain patients” (Facebook, 2,200 members). The questionnaire was posted three times between 10 and 30 March 2022.

**Results:**

207 respondents answered all questions in the survey of which 95% were women. Most of them (62 %) were between 36 and 55 years. 37 % live with chronic conditions for more that 20 years. 72 % of the respondent experience lack of continuity with the healthcare system, often with regard to information or communication with health professionals and they look for peers’ advices or experiences in the online groups. 68 % felt recognized by the peers in the online community in another way than in the meeting with the healthcare system.

**Conclusions:**

Patients use PLOC to find and exchange experiences from other patients with chronic conditions about their treatment and especially daily life with chronic conditions.

**Key messages:**

• Patients participating in peer-led online communities provide online support and recognition to each other than that provided by the healthcare system.

• Patients use peer-led online communities to read about other patients’ experiences with chronic conditions and learn about their treatment and daily life.

